# Adenosine A2A Receptor Stimulation Inhibits TCR-Induced Notch1 Activation in CD8+T-Cells

**DOI:** 10.3389/fimmu.2019.00162

**Published:** 2019-02-07

**Authors:** Claudia Sorrentino, Fokhrul Hossain, Paulo C. Rodriguez, Rosa A. Sierra, Antonio Pannuti, Stephen Hatfield, Barbara A. Osborne, Lisa M. Minter, Lucio Miele, Silvana Morello

**Affiliations:** ^1^Department of Pharmacy, University of Salerno, Fisciano, Italy; ^2^Department of Genetics and Stanley S. Scott Cancer Center, Louisiana State University Health Sciences Center, New Orleans, LA, United States; ^3^H. L. Moffitt Comprehensive Cancer Center, Tampa, FL, United States; ^4^New England Inflammation and Tissue Protection Institute, Northeastern University, Boston, MA, United States; ^5^Department of Veterinary and Animal Sciences, University of Massachusetts, Amherst, MA, United States

**Keywords:** adenosine, A2A adenosine receptor (A2AR), Notch1, CD8+T-cells, immunosuppression

## Abstract

Notch receptors signaling is required for optimal T-cell activation and function. T-cell receptor (TCR) engagement can activate Notch receptors in T-cells in a ligand-independent fashion. In this study, we examined the role of adenosine A2A receptor (A2AR) signaling pathway in regulating the activity of Notch1 induced by TCR stimulation in CD8+T-cells. A selective A2AR agonist decreased Notch1 protein expression and Notch1 cleavage, and reduced transcripts of Notch1-target genes *Hes1* and *Myc* in activated CD8+T-cells. Inhibition of TCR-induced Notch1 expression by an A2AR agonist was accompanied by increased cAMP concentration and mimicked by forskolin. This effect was associated with reduced IFN-γ and granzyme B production. The effect of an A2AR agonist was abrogated by a selective A2AR antagonist and absent in CD8+T-cells harvested from *A2AR*−/− mice. Stimulation of A2AR reduced Notch1 receptor levels by inhibiting upstream TCR signals, including ZAP70 phosphorylation, in turn impairing the generation of the active Notch1 intracellular domain (N1ICD). Direct activation of PKC with PMA and ionomycin bypassed A2AR-induced Notch1 inhibition. Overexpression of N1ICD in CD8+T-cells prevented the suppressive effects of an A2AR agonist on proliferation and cytokine release during activation. Our results identify the A2AR signaling pathway as an important regulator of TCR-induced Notch1 receptor activation in CD8+T-cells, and Notch as an important target of the immune suppressive effects of A2AR. We propose a mechanism whereby A2AR impairs CD8 T-cells function through inhibition of Notch1 receptor activation.

## Introduction

Notch signaling plays a pivotal role in the differentiation and function of several T-cell subsets [reviewed in (1–4)]. Notch proteins (Notch 1–4) are heterodimeric transmembrane receptors that are activated by engagement of transmembrane ligands (Delta-like 1, 3, 4, and Jagged 1, 2, although Delta-like 3 is likely an inhibitory ligand) (5). The interaction of Notch receptors with their ligands leads to subunit separation, followed by a trans-endocytosis of the Notch extracellular domain. This facilitates the cleavage of the Notch transmembrane subunit by metalloproteases of the ADAM family, which is followed by a second cleavage within the transmembrane domain by the γ-secretase complex. This generates the active intracellular domain of Notch (NICD) (1, 2, 6). NICD translocates into the nucleus where it associates with the transcriptional repressor CSL (CBF1-Suppressor of Hairless-LAG1), recruiting a co-activator complex, to modulate the transcription of several genes (7). In T-cells, ligand-independent activation of Notch1 receptor can be triggered through T-cell receptor (TCR) signals (8–11), through mechanisms that require receptor endocytosis. Notch1 can upregulate its own expression in T-cells (12). TCR-induced up-regulation of Notch1 expression in peripheral T-cells is associated with increased proliferation and cytokine production, including IFN-γ (8), or the expression of the transcriptional regulator eomesodermin (EOMES), which in turn regulates the expression of CD8 effectors perforin and granzyme B (10). Transgenic expression of Notch1 renders CD8 T-cells resistant to tumor-induced immune suppression (13). Pharmacological inhibitors of γ-secretase, which prevent the generation of NICD (14, 15), reduce proliferation and cytokine release from TCR-activated T-cells in a concentration-dependent manner (8, 10). Notch2 also contributes to the activity of cytotoxic T-cells, controlling directly the transcription of granzyme B, independently of EOMES (16). The expression of Notch2 can be also up-regulated during T-cell activation and can, redundantly with Notch1, modulate proliferation and IFN-γ production in CD8+T-cells (13).

Adenosine is an ATP metabolite that increases in the extracellular space in response to hypoxia and tissue injury, acting as an anti-inflammatory mediator that limits inflammation-induced damage (17). Adenosine can exert profound immunosuppression by acting on both lymphoid (18) and myeloid cells (19). The A2A receptor (A2AR) belongs to the family of adenosine receptors and is the predominant adenosine receptor type expressed in T-cells (20, 21). This is a high-affinity, Gs-coupled receptor that upon activation increases cyclic AMP (cAMP), which in turn activates protein kinase A (PKA). Stimulation of A2AR suppresses TCR signaling in a cAMP-dependent manner (22–26). Inhibition of TCR signaling by A2AR agonists is associated with reduced cytokine production, including interleukin-2 (IL-2) and interferon-γ (IFN-γ), and decreased cytotoxicity and proliferation (22–27). Selective A2AR agonists may be used for the treatment of inflammatory diseases (28). Conversely, because A2AR in tumors contributes to induce profound immunosuppression, A2AR antagonists are considered potential novel cancer immunotherapeutics (29).

Given the crucial role of A2AR signaling in limiting CD8+ T-cell responses, we evaluated its effects on Notch1 expression and signaling. We demonstrate that A2AR stimulation inhibits TCR-induced Notch1 expression and cleavage in activated CD8+T-cells, describing a novel mechanism through which adenosinergic molecules can suppress CD8+T-cells functions via A2AR.

## Materials and Methods

### CD8+ Cells Isolation and Activation

Splenocytes were collected aseptically from the spleens of naïve C57BL/6 mice (6–10 weeks old) (Charles River, Lecco, Italy) or from A2AR gene-deficient (*A2AR*−/−) C57BL/6-background mice (6–10 weeks old), kindly provided by M. Sitkovsky (Northeastern University). CD8+ T-cells were enriched with a negative selection kit (StemCell Technologies), according to manufacturer's instructions. Experiments were also performed in CD8+T-cells conditionally expressing transgenic Notch1 intracellular domain (N1IC CD8+T-cells) or floxed control cells (N1IC^f/f^ CD8+T-cells) isolated, respectively, from previously described N1IC mice and N1IC^f/f^ mice (13). Isolated cells, cultured in RPMI 1640 medium supplemented with 10% FBS, 4 mM L-Glutamine, 50 U/ml penicillin, 50 μg/ml streptomycin and 50 μM 2-mercaptoethanol, were activated in plates coated with anti-mouse CD3ε (1 μg/ml; clone 145-2C11) and anti-mouse CD28 (0.5 μg/ml; clone 37.51) antibodies up to 72 h or left unstimulated (NS). In specific sets of experiments, CD8+ T-cells were activated with 50 ng/ml phorbol 12-myristate 13-acetate (PMA) and 200 ng/ml ionomycin (both from Sigma-Aldrich) or left unstimulated (NS).

### Cell Treatments

Cells were incubated with 1 μM A2AR agonist CGS-21680 (Tocris), 1 μM A_2A_R antagonist ZM-241385 (Tocris), 1 μM γ-secretase inhibitor PF-3084014 (SelleckChem) or with 10 μM adenylate cyclase stimulator Forskolin (Sigma-Aldrich), alone or in combination, 15 min before activation with anti-CD3ε/CD28 antibodies or PMA and ionomycin. PF-3084014 was dissolved in DMSO, whilst the other agents were solubilized in PBS (vehicle). Control cells (Ctr) received vehicle before activation as described above. In specific experiments, cells were incubated with the above agents 24 h after CD3ε/CD28-stimulation.

### Proliferation Assays

To measure proliferation, 5 × 10^5^ isolated CD8+ T-cells were labeled with 1 μM carboxyfluorescein diacetate succinimidyl ester (CFDA-SE, Life Technologies) for 10 min at 37°C. Cells were then washed to remove excess CFSE and then treated with 1 μM CGS-21680, 1 μM PF-3084014, or 10 μM Forskolin before adding anti-CD3ε and anti-CD28 antibodies for 72 h. Proliferation was assessed by measuring CFSE fluorescence by flow cytometric analysis on a FACSCalibur cytometer (BD Biosciences). Data were analyzed using CellQuest software.

### ELISA Assays

Granzyme B and IFN-γ concentrations were measured in CD8+ T-cells supernatants after 72-h treatments as described above, by using specific ELISA kits (Thermo Fisher Scientific) according to the manufacturer's instructions.

### Western Blot Analysis

1 × 10^6^ cells treated as described above were collected, washed in ice-cold PBS and lysed in RIPA buffer to which were freshly added 1 mM Protease Inhibitor Cocktail (Thermo Scientific), 1 mM NaF and 1 mM sodium orthovanadate (all the reagents used for the lysis buffer were EDTA-free). Equal amounts of proteins were electrophoretically separated in SDS-Page gels and then transferred to PVDF membranes (Immobilion-FL Transfer Membrane). Bovine serum albumin (5% BSA, Sigma) was used to block non-specific binding sites. The following primary antibodies were used: anti-Notch1 (which specifically detects the Notch1 intracellular domain N1ICD, Val1744; D3B8), anti-Notch1^TM^, (to detect the transmembrane Notch1 domain, D1E11) (both from Cell Signaling), anti-pZAP70 (pY319.17A) or anti-GAPDH, anti-β-tubulin or anti-actin as controls (Santa Cruz Biothecnology). After incubation with appropriate secondary antibodies, immune-reactive protein bands were detected by enhanced chemiluminescence reagents (Amersham Pharmacia Biotech, UK) and analyzed by Las4000 (GE Healthcare Life Sciences). The optical density of protein bands detected by Western blotting was calculated with ImageQuantTL (GE Healthcare).

### Quantitative Real-Time RT-PCR

Total RNA was isolated from treated cells using an RNeasy Mini kit (Qiagen) following the manufacturer's instructions. Reverse transcription was performed using the First strand cDNA synthesis Kit (Fermentase Life Science Co.). cDNAs were amplified by real-time PCR using iTaqTM SYBR Green Supermix with ROX (Bio-RAd). Primer sequences were:

Notch1(Forward): AGCGGGGTATGCAAGGAGTC, (Reverse): CTCGCAGGTTTGACCTTGCC;
Hes1(Forward): CTGGTGCTGATAACAGCGGAATC, (Reverse): AGTGATCGGTAGCACTATTCCAGG;
Myc(Forward): GCAGATCAGCAACAACCGCA, (Reverse): CCAAGACGTTGTGTGTCCGC;
IFNγ(Forward): TGCATCTTGGCTTTGCAGCTC, (Reverse): GGCTTTCAATGAGTGTGCCGT.
Rpl3a(Forward): GGAGGGGCAGGTTCTGGTAT, (Reverse): TTCACAGCGTACGACCACCA.

Samples were run in triplicate in a 96 well Optical Reaction plate (Applied BioSystems). The PCR reaction conditions were: 50°C for 2 min, 95°C for 5 min, 35 cycles of 95°C for 30 s, 59°C for 1 min, 72°C for 1 min, followed by dissociation step. Granzyme B RT-PCR analysis was performed by using Taqman primers Mm00442837_m1 and GAPDH: Mm99999915_g1 as housekeeping gene (Applied Biosystems). Results were analyzed using the 7,300 system Software (Applied BioSystems).

### cAMP Assays

cAMP levels were measured with the cAMP assay kit (Abcam), according to the manufacturer's instructions. cAMP levels were determined in 1 × 10^6^ CD3ε/CD28-stimulated CD8+ T-cells pre-incubated with 1 μM CGS-21680 or 10 μM Forskolin. 10 min later, cells were incubated with 0.1 M HCl at room temperature for 20 min. After gentle pipetting, samples were centrifuged for 10 min and supernatants collected. Samples were then assayed for cAMP levels.

### Statistical Analysis

Unless otherwise stated, results are expressed as means ± SD. Data were analyzed with GraphPad Prism 6 (GraphPad Software). Two-tailed Student's *t*-test (2-group comparisons) or ANOVA (>2-group comparisons) were performed as appropriated, with Bonferroni correction for multiple comparisons when needed. *P* values < 0.05 were considered significant.

## Results

### A2AR Stimulation Leads to Reduced Expression and Activity of Notch1 in CD3ε/CD28-Activated CD8+T-Cells

**Figure 1:**
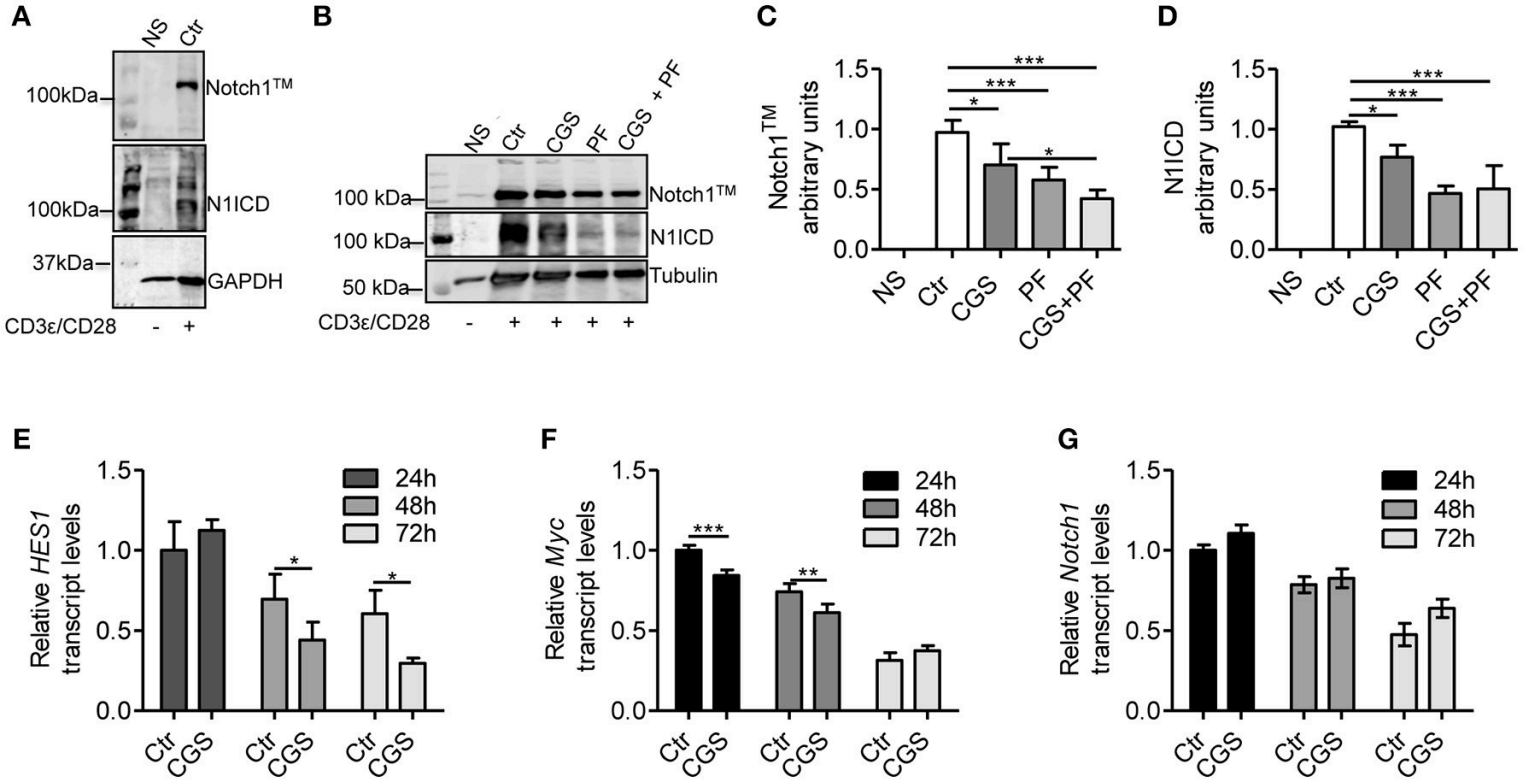
CGS-21680 inhibits TCR-induced Notch1 protein increase and reduces the expression of N1ICD target genes in CD3ε/CD28-stimulated CD8+T-cells. **(A)** Isolated splenic CD8+T-cells from C57Bl6 mice were stimulated with anti-CD3e and anti-CD28 antibodies for 72 h and whole-cell extracts were analyzed for Notch1 by Western blotting. The transmembrane, uncleaved Notch1 subunit, Notch1^TM^ (top panel) and the intracellular Notch1 domain, N1ICD (lower panel) in stimulated CD8+T-cells or unstimulated cells are shown. **(B)** Notch1 expression was examined in unstimulated CD8+T-cells (NS) or in CD8+T-cells treated with: vehicle (Ctr); A2AR agonist CGS-21680 (1 μM; CGS); GSI PF-3084014 (1 μM; PF) or both (CGS+PF) for 15 min before stimulation with anti-CD3ε and anti-CD28 antibodies. **(C,D)** Densitometry analyses of Notch1^TM^ and N1ICD, respectively, normalized against tubulin. Results represent mean ± SD from nine independent experiments. ^*^p < 0.05; ^***^p < 0.001; one-way ANOVA followed by Bonferroni correction for multiple comparisons. **(E)** HES1, **(F)** c-Myc, and **(G)** Notch1 mRNAs were measured in CD8+T-cells activated with anti-CD3ε/CD28 antibodies after CGS-21680 (1 μM) incubation, and determined at 24–48–72 h. Results represent means ± SD from three different animals, tested in triplicate. ^*^p < 0.05, ^**^p < 0.01, ^***^p < 0.001, two-way ANOVA with post Bonferroni test.

To evaluate the mechanistic interaction between A2AR and Notch signaling, we pre-incubated for 15 min mouse CD8+ T-cells with the selective A2AR agonist CGS-21680 prior to adding anti-CD3ε/CD28 specific antibodies. As Notch1 receptor proteolytic cleavage/activation is induced by TCR stimulation (8, 10, 11, 30), we evaluated the levels of Notch1 receptor proteins (the transmembrane Notch1 subunit, Notch1^TM^ and the intracellular Notch1 domain, N1ICD) in activated CD8+ T-cells compared to unstimulated cells. Activated CD8+T-cells strongly expressed Notch1^TM^ and N1ICD proteins, compared to non-stimulated (NS) counterparts (Figure 1A). Notably, incubation of CD8+T cells with CGS-21680 significantly reduced the expression of both Notch1^TM^ and N1ICD (Figures 1B–D), suggesting that A2AR activation interferes with TCR signaling. As a control, we treated cells with the γ-secretase inhibitor (GSI) PF-3084014, which potently inhibits Notch1 cleavage (31). Incubation of cells with PF-3084014 (1 μM) prevented the generation of N1ICD following anti-CD3ε/CD28 stimulation (Figures 1B–D). Cells treated with PF-3084014 alone or together with CGS-21680 showed the highest Notch 1 down-regulation (Figures 1B–D).

To further investigate the effect of the A2AR agonist on TCR-induced Notch1 signaling pathway, we determined the expression of N1ICD-target genes *Hes1* (32) and *cMyc* (33). *Hes1* and *cMyc* mRNA levels were reduced in CD8+T-cells treated with CGS-21680 (1 μM) and stimulated with anti-CD3ε/CD28 (Figures 1E,F, respectively). In particular, *Hes1* mRNA levels upon TCR stimulation were significantly reduced 48 and 72 h after CGS-21680 treatment (Figure 1E). *cMyc* mRNA levels were significantly decreased at 24 and 48 h of treatment (Figure 1F). These results suggest that stimulation of A2AR decreases the expression and activation of Notch1 and N1ICD-mediated transcriptional activity in CD3ε/CD28-stimulated CD8+T-cells. The different time courses of the two transcripts may be related to different half-lives of these two transcripts or to the different mechanisms whereby N1ICD regulates the expression of *Hes1* and *cMyc* in T-cells. *Hes1* is regulated largely through a Sequence-Paired Site (SPS) closely associated with the *Hes1* transcriptional start site (34), whereas *cMyc* is regulated primarily through a distal super-enhancer whose acetylation status is highly sensitive to depletion of N1ICD (35).

To determine whether the lower levels of Notch1 protein were due to reduced mRNA synthesis, we analyzed *Notch1* transcript levels in CD8+T-cells treated with CGS21680 (1 μM) and anti-CD3ε/CD28. *Notch1* mRNA levels were unchanged in CD8+T-cells incubated with CGS-21680 compared to control cells (Figure 1G), indicating that A2AR stimulation decreases the levels of Notch1 protein without affecting *Notch1* transcription.

### The Inhibitory Effect of CGS-21680 on TCR-Induced Notch1 Expression Depends on A2AR Stimulation

**Figure 2:**
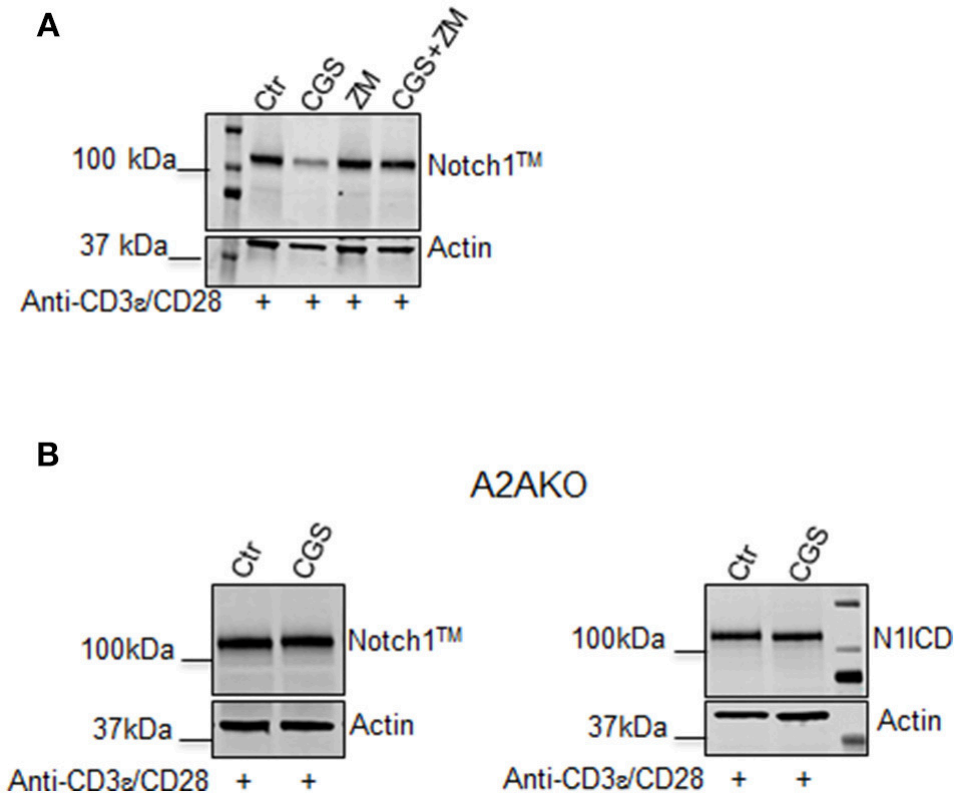
The effect of CGS-21680 on Notch1 protein levels depends on A2AR stimulation. **(A)** CD8+T-cells from the spleens of C57Bl6 mice were incubated with: CGS-21680 (1 μM; CGS); the A2AR antagonist ZM-241385 (1 μM; ZM); or both and then stimulated with anti-CD3ε/CD28 antibodies. Blot showing Notch1^TM^ is representative of three independent experiments. **(B)** CD8+T-cells isolated from the spleens of *A2AR*−/− mice were treated with vehicle (Ctr) or CGS-21680 (1 μM; CGS) before activation with anti-CD3ε/CD28 antibodies and whole-cell extracts were analyzed for Notch1^TM^ (left panel) and the intracellular Notch1 domain, N1ICD (right panel) by Western blotting.

To confirm that the effect of CGS-21680 on Notch1 expression was dependent on A2AR stimulation, we performed experiments in presence of the A2AR antagonist ZM-241385 (1 μM), administered in combination with CGS-21680 (1 μM). Treatment with ZM-241385 (1 μM) reversed the inhibitory effect of CGS-21680 on Notch1 expression in CD3ε/CD28-stimulated CD8+T-cells (Figure 2A). To rule out off-target effects induced by the CGS-21680, we treated T cells lacking *A2AR*. In *A2AR*−/− CD8+T-cells, CGS-21680 treatment did not affect TCR-induced Notch1 levels or N1ICD levels (Figure 2B), indicating that the observed CGS-21680 effects were triggered through stimulation of A2AR. No differences in the levels of Notch2, 3, or 4 were observed in *A2AR*−/− CD8+T-cells (Supplemental Figure 1).

### A2AR Stimulation and Notch1 Inhibition Strongly Reduce Proliferation and Cytokine Production in Activated CD8+ T-Cells

**Figure 3:**
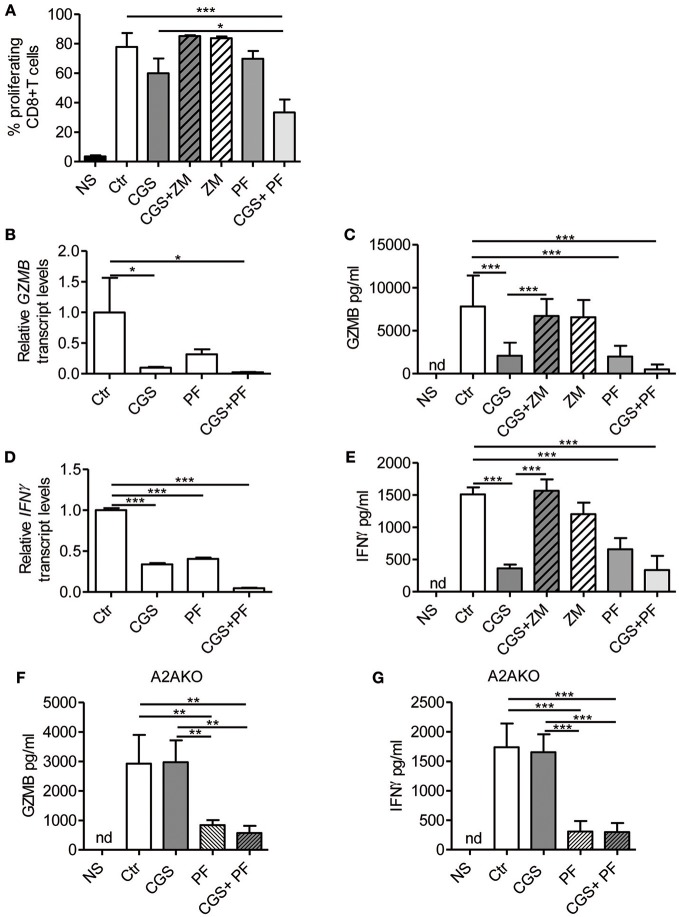
A2AR stimulation and γ-secretase inhibition decrease CD8+T-cell effector functions. **(A)** CD8+T-cells were labeled with CFSE and then incubated with CGS-21680 (1 μM; CGS), PF-3084014 (1 μM; PF) or both (CGS+PF) for 15 min before stimulation with anti-CD3ε/CD28 antibodies for 72 h. Proliferation was also assessed in cells incubated with the A2AR antagonist ZM-241385 (1 μM; ZM) alone or in combination with CGS-21680 (CGS+ZM). CFSE-labeled cells were counted by flow cytometry. Results represent means ± SD of the percentage of proliferating CD8+T-cells from six different mice, tested in duplicate. ^*^p < 0.05; ^***^p < 0.001; one-way ANOVA followed by Bonferroni correction for multiple comparisons. **(B)** Expression of granzyme B (GZMB) mRNA was determined by real-time RT-PCR in CD8+T-cells pre-treated with CGS and/or PF before anti-CD3ε/CD28 antibodies stimulation for 24 h; **(C)** granzyme B was quantified by ELISA in supernatants harvested from cells treated as described in **(A)** for 72 h. Results are means ± SD from three independent experiments. ^*^p < 0.05; ^***^p < 0.001. **(D)** Expression of interferon-γ (IFN- γ) mRNA was determined by real-time RT-PCR in CD8+T-cells pre-treated with CGS and/or PF before anti-CD3ε/CD28 antibodies stimulation for 24 h and **(E)** IFN-γ was quantified by ELISA in supernatants harvested from cells treated as described in **(A)** for 72 h. Results represent mean ± SD from three independent experiments. ^***^p < 0.001; one-way ANOVA followed by Bonferroni correction for multiple comparisons. **(F,G)** levels of granzyme B and IFN-γ, respectively, in supernatants harvested from CD8+T-cells isolated from spleen of *A2AR*−/− mice (*n* = 4) and pre-treated with CGS and/or PF before anti-CD3ε/CD28 antibodies stimulation for 72 h. Results represent means ± SD. ^**^p < 0.01; ^***^p < 0.001; one-way ANOVA followed by Bonferroni correction for multiple comparisons. Unstimulated cells (NS) and vehicle-treated cells (Ctr) were used as controls. Nd, not determined.

Stimulation of A2AR strongly inhibits the proliferation (22) and the effector functions of activated T-cells, including cytokine production (23–27). On the other hand, Notch1 activation promotes proliferation (8) and cytokine release (8, 10, 36) in CD8+T-cells. We therefore investigated the cross-talk between these signaling pathways in regulating the functional properties of CD8+T-cells, by measuring proliferation and cytokine levels in cells treated with CGS-21680 alone or in combination with GSI PF-3084014 before TCR-activation. Proliferation was assessed by flow cytometry analysis after CFSE staining. The increased CD8+ T-cell proliferation induced upon stimulation with anti-CD3ε/CD28 was slightly reduced after pre-incubation with CGS-21680 or PF-3084014 (Figure 3A). Moreover, an additive anti-proliferative effect was observed after the combination of CGS-21680 plus GSI PF-3084014 (Figure 3A).

Experiments were also performed in cells treated with the A2AR antagonist ZM-241385 (1 μM). Further inhibition of A2AR signaling through ZM-241385 did not affect proliferation in anti-CD3ε/CD28-activated T cells (Figure 3A).

Transcript levels of granzyme B and IFN-γ were significantly reduced in cells treated with 1 μM CGS-21680 or 1 μM PF-3084014 and even more so after combination treatment with CGS-21680 + GSI before stimulation with anti-CD3ε/CD28 antibodies for 24 h (Figures 3B,D, respectively). Consistent with mRNA levels, the amounts of granzyme B and IFN-γ released from activated CD8+T-cells treated with CGS-21680 (1μM) and/or PF-3084014 (1 μM) for 72 h were significantly reduced compared to control cells (Figures 3C,E, respectively). These data indicate that A2AR stimulation and γ-secretase inhibition strongly impair the functions of activated CD8+T-cells. The effects of GCS-21680 on both granzyme B and IFN-γ were completely reversed by the A2AR antagonist ZM-241385 (1 μM) (Figures 3C,E, respectively).

Treatment of *A2AR*−/− CD8+T-cells with CGS-21680 (1 μM) failed to reduce the release of either granzyme B (Figure 3F) or IFN-γ (Figure 3G), confirming that the suppressive effect of CGS-21680 on the release of these factors was completely dependent upon the presence of A2AR. Conversely, inhibition of γ-secretase with PF-3084014 led to a significant reduction of both granzyme B and IFN-γ release from activated CD8+T-cells from *A2AR*−/− mice (Figures 3F,G, respectively). These data indicate that Notch effects on granzyme B and IFN-γ release are independent of A2AR.

### Adenylate Cyclase Stimulation Mimics the Effects of CGS-21680 on Notch1 in Activated CD8+ T-Cells

**Figure 4:**
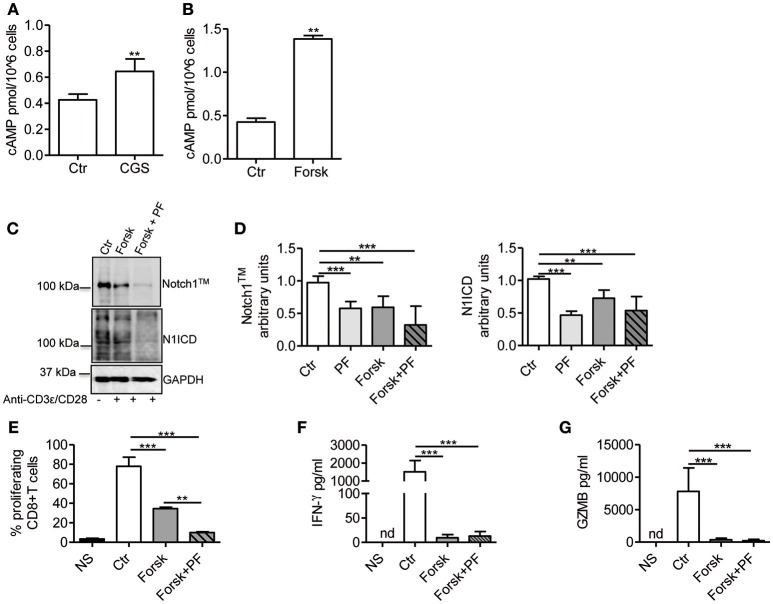
cAMP inhibits TCR-induced Notch1 activation in CD8+T-cells. Levels of cAMP were measured in cells incubated with CGS-21680 (1 μM; CGS) **(A)** or Forskolin (10 μM; Forsk) **(B)** before stimulation with anti-CD3ε/CD28 antibodies for 10 min. Activated CD8+T-cells (Ctr) were used as controls. Results represent means ± SD from four different animals, tested in duplicates. ^**^p < 0.01; Mann-Whitney test. **(C)** Protein levels of Notch1^TM^ (upper panel) and N1ICD (lower panel) in CD8+T-cells pre-treated with Forskolin (10 μM; Forsk) alone or in combination with GSI PF-3084014 (1 μM; PF) before activation with anti-CD3ε/CD28 antibodies for 72 h. Unstimulated cells (NS) or activated cells (Ctr) were used as controls. **(D)** Densitometric analyses of Notch1^TM^ (left panel) and N1ICD (right panel) normalized against GAPDH. Results represent means ± SD from four independent experiments. ^**^p < 0.01; ^***^p < 0.001; one-way ANOVA followed by Bonferroni correction for multiple comparisons. **(E)** Percentage of proliferating CD8+T-cells treated as described in C determined by flow cytometry after CFSE staining. Results represent means ± SD from three independent experiments. ^**^p < 0.01; ^***^p < 0.001; one-way ANOVA followed by Bonferroni correction for multiple comparisons. Levels of IFN-γ **(F)** and granzyme B (GZMB) **(G)** measured by ELISA in supernatants collected from cells treated as described in **(C)**. Results represent mean ± SD from four independent experiments. ^***^p < 0.001; one-way ANOVA followed by Bonferroni correction for multiple comparisons. Nd, not determined.

Stimulation of A2AR increases intracellular levels of cAMP in activated T-cells (22, 26). Consistent with these reports, we observed that CGS-21680 treatment (1 μM) of CD3ε/CD28-stimulated CD8+ T-cells increased cAMP levels within 10 min (Figure 4A). To evaluate whether increased levels of cAMP affected TCR-induced Notch1 signaling, we used the adenylate cyclase activator Forskolin (10 μM), which significantly increased cAMP levels in activated T-cells (Figure 4B). Forskolin significantly decreased the expression of Notch1 proteins induced by TCR engagement in CD8+T-cells, proliferation, production of IFN-γ and granzyme B (Figures 4C–G). When we combined Forskolin and GSI PF-3084014 (Figures 4C,D), the effect on Notch1 proteins was marked. These results suggest that cAMP-elevating agents can markedly decrease the amount of Notch1 in activated CD8+ T-cells and significantly impair Notch-dependent functions of TCR-stimulated CD8+T-cells.

### Ectopic Expression of N1IC Rescues CD8+ T-Cells From the Suppressive Effects of CGS-21680

**Figure 5:**
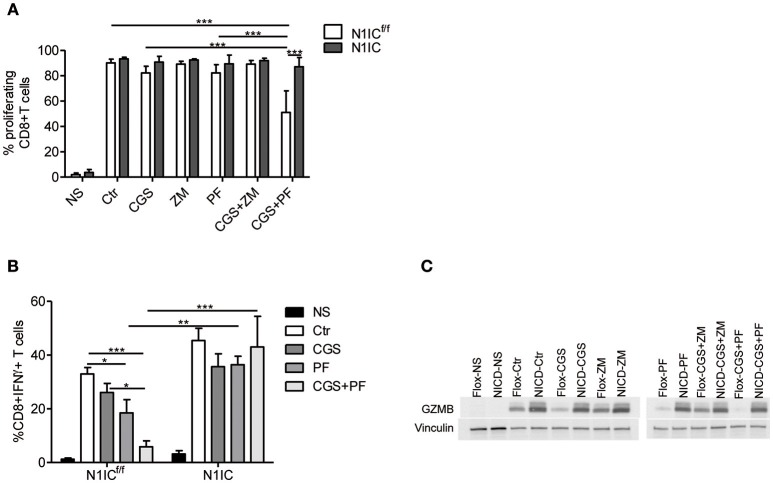
Transgenic expression of Notch1 ICD abrogates the suppressive effects of CGS-21680 on CD8+T-cells functions. **(A)** CD8+T-cells isolated from spleen of N1IC^f/f^ or N1IC mice were activated with anti-CD3/CD28 antibodies and 24 h later cells were labeled with CFSE and incubated in presence or absence of: CGS-21680 (1 μM; CGS); PF-3084014 (1 μM; PF); CGS in combination with PF (CGS+PF); or ZM-241385 (1 μM; ZM) alone or in combination with CGS. CD8+T-cells proliferation was assessed after 72 h by flow cytometry. Results represents means ± SD of the percentage of proliferating cells from three independent experiments. ^***^p < 0.001; two-way ANOVA followed by Bonferroni correction for multiple comparisons. **(B)** Expression of IFN-γ was assessed in cells stimulated as described in **(A)**. The percentage of CD8+IFN-γ+T-cells was determined by flow cytometry. Results represent means ± SD from four independent experiments. ^*^p < 0.05; ^**^p < 0.01; ^***^p < 0.001; two-way ANOVA followed by Bonferroni correction for multiple comparisons. **(C)** CD8+T-cells from N1IC^f/f^ or N1IC mice were stimulated as described in **(A)**, whole-cells extracts were collected and expression of granzyme B (GZMB) was determined by Western blotting. Blot representative of two independent experiments is shown.

To determine the functional role of the inhibition of Notch1 signaling in the suppressive effects induced by A2AR activation, we studied CD8+T-cells from activated-CD8+T cell conditional N1IC mice (13), which over-express Notch1IC upon activation. N1IC mice were created after crossing N1IC^f/f^ mice with those expressing Cre Recombinase driven by Granzyme B promoter. Activated N1IC CD8+ T-cells or floxed controls (N1IC^f/f^) were treated with CGS-21680 and/or GSI PF-3084014 and evaluated for proliferation or IFN-γ and granzyme B expression. Similar to the results observed in wild-type T cells, treatment of N1IC^f/f^ cells with CGS-21680 and/or PF-03084014 slightly reduced the proliferation (Figure 5A). Conversely, activated N1IC CD8+ T-cells showed no restriction in their proliferation after treatment with CGS-21680 or PF-3084014 or a combination of both (Figure 5A), suggesting that restoring Notch1 signaling in CD8+T-cells prevents the suppressive effects induced by A2AR activation.

Next, we compared the expression of IFN-γ and granzyme B in N1IC vs. N1IC^f/f^ CD8+ T-cells treated with CGS-21680, GSI PF-3084014 or both. In agreement with the key role of Notch in the effects induced by A2AR, we found that while CGS-21680 and/or PF-3084014 reduced the frequency of IFN-γ-expressing cells and the expression of granzyme B in control N1IC^f/f^ CD8+ T-cells, they had no significant effect in N1IC CD8+ T-cells (Figures 5B,C). Overall, these results indicate that inducible expression of Notch1 intracellular domain is sufficient to overcome the suppressive effects of A2AR agonists in activated CD8+T-cells, and support the hypothesis that Notch1 is a functionally important target of A2AR-mediated suppressive effects in these cells.

### A2AR Stimulation Inhibits TCR-Induced Notch1 Activation/Expression by Blocking Proximal TCR Events

**Figure 6:**
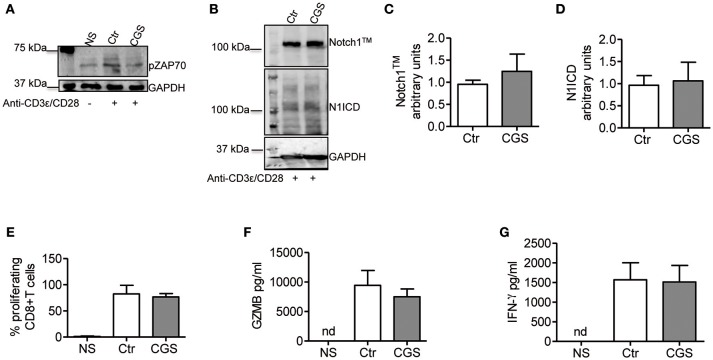
CGS-21680 inhibits the early events of TCR signal transduction. **(A)** ZAP70 phosphorylation was examined in CD8+T-cells lysates after 10 min stimulation with anti-CD3/CD28 antibodies of untreated cells (Ctr) or CGS-21680-pretreated cells (1 μM; CGS). Unstimulated cells (NS) were used as controls. One experiment representative of three independent experiments is shown. **(B)** Expression of Notch1^TM^ (upper panel) and N1ICD (lower panel) at 72 h in CD8+T-cells stimulated with anti-CD3ε/CD28 antibodies for 72 h. Cells were treated with CGS-21680 (1 μM; CGS) or vehicle (Ctr) 24 h after anti-CD3ε/CD28 stimulation. Densitometric analyses of Notch1^TM^ **(C)** and N1ICD **(D)** bands normalized against GAPDH. Results represent means ± SD from four independent experiments. **(E)** Percentage of proliferating CD8+T-cells labeled with CFSE, treated as described in **(B)** as determined by flow cytometry. Results represent means ± SD from four independent experiments. Levels of granzyme B (GZMB) **(F)** and interferon-gamma **(G)** measured by ELISA in supernatants collected from cells treated as described in **(B)**. Results represent means ± SD from five independent experiments. Nd, not determined.

In CD4+ T-cells, TCR stimulation triggers Notch1 activation within 2 h with a peak at 6 h (11). This activation is mediated by a unique, Notch ligand-independent process that requires Notch endocytosis and signals from the TCR through Lck, ZAP70, phospholipase C gamma (PLCγ), a diacylglycerol-activated protein kinase C (presumably PKCθ), Ca^++^ influx as well as ADAM10 or 17 and γ-secretase. PKC is activated by PDK1, in response to a PI3K co-activating signal from CD28 (37). We reasoned that if Notch1 activation in CD8+ T-cells is mediated by a similar mechanism, A2AR activation would suppress ZAP70 phosphorylation, while activation of PKC and Ca^++^ influx downstream of ZAP70 would bypass A2AR-mediated Notch inhibition. Additionally, we hypothesized that A2AR agonists would be unable to block Notch1 if given after early events in TCR activation have occurred. A2AR stimulation inhibits early TCR signaling by increasing the levels of cAMP during T-cell activation. This effect is mediated by reduced ZAP70 phosphorylation (26, 38). Consistent with these observations, we found that incubation of cells with CGS-21680 (1 μM) reduced Tyr319 ZAP70 phosphorylation in activated CD8+T-cells (Figure 6A). Treatment of CD8+ T-cells cells with CGS-21680 24 h after incubation with anti-CD3ε/CD28 antibodies had no effect on Notch1 protein levels (Figures 6B–D). Proliferation and release of granzyme B and IFN-γ from activated CD8+T-cells were unchanged by CGS-21680 treatment once the early TCR signal had been transduced (Figures 6E–G, respectively).

**Figure 7:**
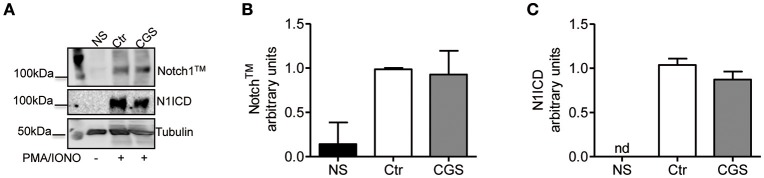
Stimulation of A2AR does not inhibit the activation of Notch1 in PMA/Ionomycin-stimulated CD8+T-cells. **(A)** quantification and densitometric analysis of Notch1^TM^ **(B)** and N1ICD **(C)** proteins in whole-cell lysates from CD8+T-cells pre-treated with CGS-21680 (1 μM; CGS) for 15 min and then stimulated with PMA/Ionomycin for 24 h. Unstimulated cells (NS) and untreated cells (Ctr) were used as controls. Results represent means ± SD from three independent experiments. Nd, not determined.

To determine whether A2AR agonists affect later stages of the TCR signaling cascade leading to Notch1 activation, we activated CD8+ T-cells with PMA and ionomycin. PMA and ionomycin induce protein kinase C (PKC) activity and Ca^++^ influx (39). CD8+T-cells activated with PMA and ionomycin showed much higher levels of Notch1^TM^ and N1ICD compared to unstimulated cells (Figures 7A–C). In addition, treatment of these cells with CGS-21680 (1 μM) before stimulation with PMA and ionomycin had no effect on Notch1 protein levels (Figures 7A–C), suggesting that A2AR acts upstream of PKC activation during TCR-induced Notch activation. Taken together, these results indicate that the suppressive effects of CGS-21680 through A2AR on Notch1 activation rely in its capacity to block early TCR signaling events upstream of PKC activation, most likely ZAP70 phosphorylation. These effects are lost once TCR signals have been transduced and the first wave of N1ICD generation has occurred.

## Discussion

In the present study we describe a previously unknown role for the A2A adenosine receptor pathway in the control of TCR-induced Notch1 receptor activation in CD8+T-cells. Stimulation of A2AR decreases the levels of Notch1 receptor protein stabilized by TCR ligation, as well as the levels of cleaved active intracellular domain generated in CD3ε/CD28-stimulated CD8+ T-cells. This effect requires A2AR and is mediated by inhibition of TCR signaling upstream of PKC activation.

**Figure 8:**
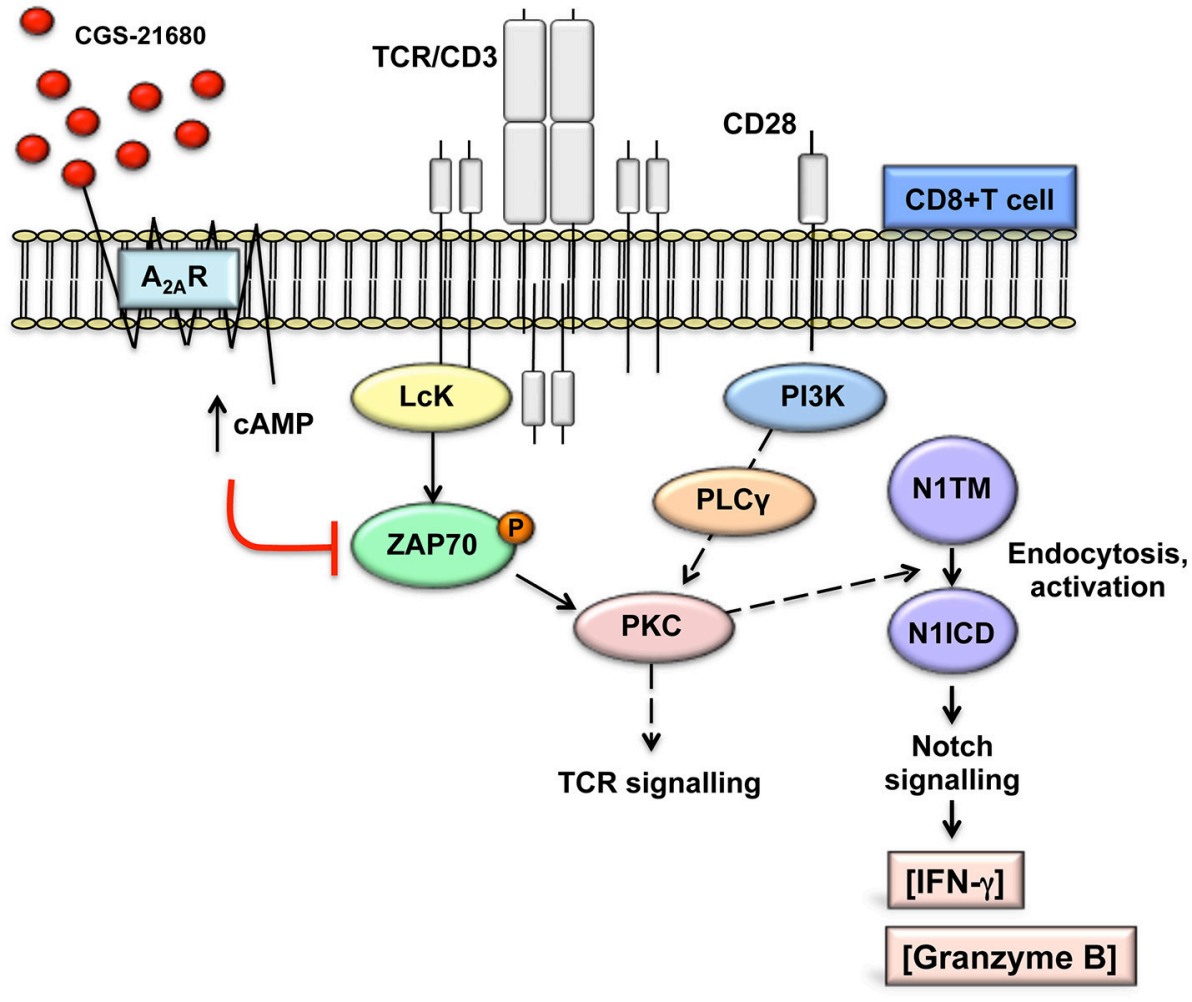
Working model: A2AR stimulation prevents TCR-induced generation of Notch1 intracellular domain by attenuating early TCR signaling events in a cAMP-dependent manner. This prevents PKC-induced Notch1 endosomal activation. Since no Notch ligands were used in our experimental system, we propose that Notch1 activation follows a ligand-independent, endosomal mechanism as described in CD4+ T-cells. The PKC isoform involved has not been determined. In turn, reduced Notch1 signaling results in decreased expression of direct and indirect Notch target genes, such as granzyme B and INF-γ, thereby impairing the effector functions of activated CD8+T-cells. This model is consistent with our observations that direct stimulation of PKC-induced Notch1 activation with PMA and ionomycin bypasses cAMP-mediated inhibition and that inducible expression of N1ICD abrogates the effects of A2AR stimulation. We have not determined whether cAMP causes Notch1 to be targeted for lysosomal degradation.

Consistent with published results (22, 26), activation of A2AR increases intracellular levels of cAMP. cAMP-dependent protein kinase A (PKA) phosphorylates and activates the C-terminal SRC kinase (CSK), which in turn phosphorylates and inactivates Lck, reducing tyrosine phosphorylation of the TCR/CD3ζ chain during T-cells activation (40). Lck-mediated tyrosine phosphorylation of the TCR/CD3 ζ chain after T-cell activation is required for the recruitment to CD3 and activation of the zeta-chain-associated protein kinase 70 (ZAP70) and subsequent activation of other substrates that initiate downstream signals (40). In line with previous data (26, 41), stimulation of A2AR with CGS-21680 reduced ZAP70 phosphorylation. Therefore, we propose a mechanism whereby stimulation of A2AR, by attenuating early TCR transduction signaling in a cAMP-dependent manner, can prevent the generation of the Notch1 intracellular domain (N1ICD) induced by TCR signaling in CD8+T-cells (Figure 8). This working model could apply not only to A2AR but also potentially to other immune suppressive molecules that signal through cAMP and PKA in T-cells, such as prostaglandin E2 (42, 43).

Our data indicate that A2AR signals act upstream of a diacylglycerol-dependent PKC and Ca^++^, as PMA and ionomycin bypass the effect of A2AR activation upon Notch1. Recent data show that TCR-induced, Notch ligand-independent Notch1 cleavage in T-cells requires the activation of one or more PKC isoforms, which facilitate ADAM activity (11). Previous reports indicate that the activities of ADAM10 and 17, involved in the S2 Notch1 cleavage (44) are regulated by PKCα and PKCθ (45, 46). Activation of PKC is a downstream event of the TCR signaling, which depends on diacylglycerol produced by phospholipase C (PLC)-γ. PLC-γ activation in turn is triggered by the phosphorylation of ZAP70 (47). The stable activation and membrane translocation of PKCθ requires Vav and phosphatidylinositol 3-kinase (PI3K) activation, triggered by the CD28 costimulatory signal, in addition to TCR/CD3 signal (47). PKCθ, a Ca^++^-independent, phospholipid-dependent PKC isoform, is a central player in the immunological synapse, responsible for the activation of AP-1 and NF–κB downstream of the TCR (48). In this study, we observed that activation of CD8+T-cells with PMA and ionomycin, which directly and potently stimulate the activity of PKCs (47), increases the levels of Notch1 and consequently its activity. This is similar to what was described by Steinbuck and collaborators (11) in CD4+ T-cells. Our data indicate that the process of Notch1 activation in CD8+ T-cells is likely similar to what these authors described, involving a ZAP70-dependent, ligand-independent endocytosis process that results in Notch internalization and cleavage in the endosomal compartment. Notch endocytosis can result in either endosomal γ-secretase cleavage and release of N1ICD into the cytoplasm or lysosomal degradation of uncleaved receptor. E3 ubiquitin ligases Deltex (DTX1 in mammals) and Suppressor of Deltex (*Itchy* in mice and AIP4 in humans) target Notch1 to endosomal activation or lysosomal degradation, respectively [see (49, 50) for reviews]. In many mammalian cells, inactive Notch receptors are constitutively targeted to lysosomal degradation via Itchy/AIP4 (50). In T-cells, E3 ligase Cbl ubiquitinates Notch1 and Notch3 and targets them to lysosomal degradation (51, 52). Interestingly, PKCθ phosphorylates and inactivates Cbl (47), and PI3K-AKT protects Notch1 from Cbl-induced degradation in T-ALL (51). The fact that in our cells Notch1 protein was virtually undetectable at rest but rapidly increased and was cleaved to generate N1ICD upon TCR activation suggests that TCR signals in CD8+ T-cells can shift Notch1 protein from a default rapid lysosomal degradation pathway, perhaps mediated by Cbl-catalyzed ubiquitination, to an endosomal activation pathway, and that A2AR modulates this process via cAMP. Further investigations on these mechanisms may help to better understand how adenosine, and possibly other cAMP-generating immune suppressive mediators, regulate Notch1 activity in CD8+T-cells.

The effect of CGS-21680 on Notch1 expression is A2AR-dependent, since it was completely reversed by a selective A2AR antagonist and lost in CD8+T-cells from *A2AR*−/− mice. A2AR activation with CGS-21680 markedly inhibits the production of IFN-γ and granzyme B released from CD3ε/CD28-stimulated CD8+T-cells. These data are consistent with previous studies describing a critical role of A2AR in controlling CD8+T-cell activation in a cAMP-dependent manner, by suppressing proximal TCR signaling events (18, 53). These effects as well were completely lost in activated CD8+T-cells from *A2AR*−/− mice, as well as in wild-type cells incubated with CGS-21680 after TCR stimulation.

In CD4+ T-cells, Notch augments NF-κB activity after TCR activation (36). Moreover, Notch1 is required for CD8 effector functions, including production of IFN-γ and granzyme B (10). GSIs suppress production of IFN-γ and granzyme B (30). Consistent with published observations, GSI PF-3084014 did decrease production of IFN-γ and granzyme B in CD8+ T-cells. Our data show that simultaneous inhibition of Notch and stimulation of A2AR profoundly impair the responses of activated CD8+T-cells, including proliferation and cytokine production. This suggests that in tumor microenvironments rich in adenosine and other immune-suppressive mediators, GSIs may suppress anti-tumor CD8+ responses. GSIs have multiple targets (14), including Notch2, which is also induced by TCR stimulation in T-cells although to a lesser extent than Notch1 (54). Our data show that CD8+ T-cells ectopically expressing N1IC are remarkably resistant to the inhibitory effects of A2AR stimulation compared with controls N1IC^f/f^ cells. These results strongly indicate that intracellular levels of Notch1 regulate the sensitivity of CD8+ T-cells to A2AR agonists, and that inhibition of Notch1 is a functionally relevant mechanism whereby A2AR agonists suppress CD8+ T-cell activation. Whether A2AR antagonists can protect Notch activity in CD8+ T-cells in tumors remains to be determined. Further investigations are also required to understand whether the A2AR signaling suppresses responses in other T-cell subsets, including CD4+T-cells and memory CD4+ or CD8+ T-cells, through inhibition of Notch.

Altogether, our data suggest a new role for A2AR in blunting Notch activity, which is essential to the effector function of CD8+T-cells. We propose that the inhibitory effects of CGS-21680 on activated CD8+ T-cells are, at least partially, dependent on reduced Notch1 protein and activity after TCR stimulation. This novel cross-talk between two critical pathways offers new insights into the mechanisms whereby elevated adenosine concentrations can hamper immune cell activation in tumors and/or in inflamed injured tissues.

## Ethics Statement

All animal studies were carried out in Association for Assessment and Accreditation of Laboratory Animal Care (AAALAC)—accredited facilities in the context of protocols approved by the Institutional Animal Care and Use Committees of LSUHSC-New Orleans, the Moffitt Comprehensive Cancer Center and Northeastern University, respectively.

## Author Contributions

SM and LM conceived the study, supervised experiments, and prepared the final version of the manuscript. CS, FH, AP, and RS performed experiments and interpreted their results. PR supervised experiments in Notch1IC-transgenic T-cells, which were performed by RS. SH provided *A2AR*−/− T cells and was responsible for maintaining adenosine receptor transgenic mouse lines. BO and LMM provided expertise on TCR signaling and PKC activity, contributed to result interpretation, reviewed and edited the final version of the manuscript.

### Conflict of Interest Statement

The authors declare that the research was conducted in the absence of any commercial or financial relationships that could be construed as a potential conflict of interest.

## References

[1] Osborne BA Minter LM. Notch signalling during peripheral T-cell activation and differentiation. Nat Rev Immunol. (2007) 7:64–75. 10.1038/nri1998 17170755

[2] Radtke F Fasnacht N Macdonald HR. Notch signaling in the immune system. Immunity (2010) 32:14–27. 10.1016/j.immuni.2010.01.004 20152168

[3] Amsen D Helbig C Backer RA. Notch in T cell differentiation: all things considered. Trends Immunol. (2015) 36:802–14. 10.1016/j.it.2015.10.007 26617322

[4] Tindemans I Peeters MJW Hendriks RW. Notch signaling in T helper cell subsets: instructor or unbiased amplifier? Front Immunol. (2017) 8:419. 10.3389/fimmu.2017.00419 28458667 PMC5394483

[5] Chillakuri CR Sheppard D Lea SM Handford PA. Notch receptor-ligand binding and activation: insights from molecular studies. Semin Cell Dev Biol. (2012) 23:421–8. 10.1016/j.semcdb.2012.01.009 22326375 PMC3415683

[6] Bray SJ. Notch signalling: a simple pathway becomes complex. Nat Rev Mol Cell Biol. (2006) 7:678–89. 10.1038/nrm2009 16921404

[7] Borggrefe T Oswald F. The Notch signaling pathway: transcriptional regulation at Notch target genes. Cell Mol Life Sci. (2009) 66:1631–46. 10.1007/s00018-009-8668-7 19165418 PMC11115614

[8] Palaga T Miele L Golde TE Osborne BA. TCR-mediated Notch signaling regulates proliferation and IFN-gamma production in peripheral T cells. J Immunol. (2003) 171:3019–24. 10.4049/jimmunol.171.6.3019 12960327

[9] Adler SH Chiffoleau E Xu L Dalton NM Burg JM Wells AD. Notch signaling augments T cell responsiveness by enhancing CD25 expression. J Immunol. (2003) 171:2896–903. 10.4049/jimmunol.171.6.2896 12960312

[10] Cho OH Shin HM Miele L Golde TE Fauq A Minter LM. Notch regulates cytolytic effector function in CD8+ T cells. J Immunol. (2009) 182:3380–9. 10.4049/jimmunol.0802598 19265115 PMC4374745

[11] Steinbuck MP Arakcheeva K Winandy S. Novel TCR-mediated mechanisms of notch activation and signaling. J Immunol. (2018) 200:997–1007. 10.4049/jimmunol.1700070 29288204 PMC5854196

[12] Deftos ML He YW Ojala EW Bevan MJ. Correlating notch signaling with thymocyte maturation. Immunity (1998) 9:777–86. 10.1016/S1074-7613(00)80643-3 9881968 PMC2789684

[13] Sierra RA Thevenot P Raber PL Cui Y Parsons C Ochoa AC. Rescue of notch-1 signaling in antigen-specific CD8+ T-cells overcomes tumor-induced T-cell suppression and enhances immunotherapy in cancer. Cancer Immunol Res. (2014) 2:800–11. 10.1158/2326-6066.CIR-14-0021 24830414 PMC4125513

[14] Kopan R Ilagan MX. Gamma-secretase: proteasome of the membrane? Nat Rev Mol Cell Biol. (2004) 5:499–504. 10.1038/nrm1406 15173829

[15] Golde TE Koo EH Felsenstein KM Osborne BA Miele L. γ-Secretase inhibitors and modulators. Biochim Biophys Acta. (2013) 1828:2898–907. 10.1016/j.bbamem.2013.06.005 23791707 PMC3857966

[16] Maekawa Y Minato Y Ishifune C Kurihara T Kitamura A Kojima H. Notch2 integrates signaling by the transcription factors RBP-J and CREB1 to promote T cell cytotoxicity. Nat Immunol. (2008) 9:1140–7. 10.1038/ni.1649 18724371

[17] Ohta A Sitkovsky M. Role of G-protein-coupled adenosine receptors in downregulation of inflammation and protection from tissue damage. Nature (2001) 414:916–20. 10.1038/414916a 11780065

[18] Linden J Cekic C. Regulation of lymphocyte function by adenosine. Arterioscler Thromb Vasc Biol. (2012) 32:2097–103. 10.1161/ATVBAHA.111.226837 22772752 PMC4476649

[19] Morello S Pinto A Blandizzi C Antonioli L. Myeloid cells in the tumor microenvironment: role of adenosine. Oncoimmunology (2015) 5:e1108515. 10.1080/2162402X.2015.1108515 27141365 PMC4839347

[20] Koshiba M Rosin DL Hayashi N Linden J Sitkovsky MV. Patterns of A2A extracellular adenosine receptor expression in different functional subsets of human peripheral T cells: flow cytometry studies with anti-A2A receptor monoclonal antibodies. Mol Pharmacol. (1999) 55:614–24. 10051547

[21] Lukashev DE Smith PT Caldwell CC Ohta A Apasov SG Sitkovsky MV. Analysis of A2a receptor-deficient mice reveals no significant compensatory increases in the expression of A2b, A1, and A3 adenosine receptors in lymphoid organs. Biochem Pharmacol. (2003) 65:2081–90. 10.1016/S0006-2952(03)00158-8 12787889

[22] Huang S Apasov S Koshiba M Sitkovsky M. Role of A2a extracellular adenosine receptor-mediated signaling in adenosine-mediated inhibition of T-cell activation and expansion. Blood (1997) 90:1600–10. 9269779

[23] Koshiba M Kojima H Huang S Apasov S Sitkovsky M. Memory of extracellular adenosine A2A purinergic receptor-mediated signaling in murine T-cells. J Biol Chem. (1997) 272:25881–9. 10.1074/jbc.272.41.25881 9325320

[24] Lappas CM Rieger JM Linden J. A2A adenosine receptor induction inhibits IFN-gamma production in murine CD4+ T-cells. J Immunol. (2005) 174:1073–80. 10.4049/jimmunol.174.2.1073 15634932

[25] Ohta A Kjaergaard J Sharma S Mohsin M Goel N Madasu M. *in vitro* induction of T cells that are resistant to A2 adenosine receptor-mediated immunosuppression. Br J Pharmacol. (2009) 156:297–306. 10.1111/j.1476-5381.2008.00019.x 19076726 PMC2697837

[26] Linnemann C Schildberg FA Schurich A Diehl L Hegenbarth SI Endl E Adenosine regulates cd8 t-cell priming by inhibition of membrane-proximal t-cell receptor signaling. Immunology (2009) 128:e728–37. 10.1111/j.1365-2567.2009.03075.x 19740334 PMC2753927

[27] Erdmann AA Gao ZG Jung U Foley J Borenstein T Jacobson KA. Activation of Th1 and Tc1 cell adenosine A2A receptors directly inhibits IL-2 secretion in vitro and IL-2-driven expansion *in vivo*. Blood (2005) 105:4707–14. 10.1182/blood-2004-04-1407 15746085 PMC1895001

[28] Antonioli L Csóka B Fornai M Colucci R Kókai E Blandizzi C. Adenosine and inflammation: what's new on the horizon? Drug Discov Today (2014) 19:1051–68. 10.1016/j.drudis.2014.02.010 24607729

[29] Leone RD Lo YC Powell JD. A2aR antagonists: Next generation checkpoint blockade for cancer immunotherapy. Comput Struct Biotechnol J. (2015) 13:265–72. 10.1016/j.csbj.2015.03.008 25941561 PMC4415113

[30] Minter LM Turley DM Das P Shin HM Joshi I Lawlor RG. Inhibitors of gamma-secretase block *in vivo* and *in vitro* T helper type 1 polarization by preventing Notch upregulation of Tbx21. Nat Immunol. (2005) 6:680–810. 10.1038/ni1209x 15991363

[31] Ran Y Hossain F Pannuti A Lessard CB Ladd GZ Jung JI. γ-Secretase inhibitors in cancer clinical trials are pharmacologically and functionally distinct. EMBO Mol Med. (2017) 9:950–66. 10.15252/emmm.201607265 28539479 PMC5494507

[32] Jarriault S Le Bail O Hirsinger E Pourquie O Logeat F Strong CF. Delta-1 activation of notch-1 signaling results in HES-1 transactivation. Mol Cell Biol. (1998) 18:7423–31. 10.1128/MCB.18.12.7423 9819428 PMC109323

[33] Palomero T Lim WK Odom DT Sulis ML Real PJ Margolin A. NOTCH1 directly regulates c-MYC and activates a feed-forward-loop transcriptional network promoting leukemic cell growth. Proc Natl Acad Sci USA. (2006) 103:18261–6. 10.1073/pnas.0606108103 17114293 PMC1838740

[34] Severson E Arnett KL Wang H Zang C Taing L Liu H. Genome-wide identification and characterization of Notch transcription complex-binding sequence-paired sites in leukemia cells. Sci Signal. (2017) 10:1598. 10.1126/scisignal.aag1598 28465412 PMC5931361

[35] Yashiro-Ohtani Y Wang H Zang C Arnett KL Bailis W Ho Y. Long-range enhancer activity determines Myc sensitivity to Notch inhibitors in T cell leukemia. Proc Natl Acad Sci USA. (2014) 111:E4946–53. 10.1073/pnas.1407079111 25369933 PMC4246292

[36] Shin HM Minter LM Cho OH Gottipati S Fauq AH Golde TE. Notch1 augments NF-kappaB activity by facilitating its nuclear retention. EMBO J. (2006) 25:129–38. 10.1038/sj.emboj.7600902 16319921 PMC1356346

[37] Wang X Chuang HC Li JP Tan TH. Regulation of PKC-θ function by phosphorylation in T cell receptor signaling. Front Immunol. (2012) 3:197. 10.3389/fimmu.2012.00197 22798961 PMC3393885

[38] Sevigny CP Li L Awad AS Huang L McDuffie M Linden J. Activation of adenosine 2A receptors attenuates allograft rejection and alloantigen recognition. J Immunol. (2007) 178:4240–9. 10.4049/jimmunol.178.7.4240 17371980

[39] Chatila T Silverman L Miller R Geha R. Mechanisms of T cell activation by the calcium ionophore ionomycin. J Immunol. (1989) 143:1283–39. 2545785

[40] Vang T Torgersen KM Sundvold V Saxena M Levy FO Skålhegg BS. Activation of the COOH-terminal Src kinase (Csk) by cAMP-dependent protein kinase inhibits signaling through the T cell receptor. J Exp Med. (2001) 193:497–507. 10.1084/jem.193.4.497 11181701 PMC2195911

[41] Raskovalova T Lokshin A Huang X Su Y Mandic M Zarour HM. Inhibition of cytokine production and cytotoxic activity of human antimelanoma specific CD8+ and CD4+ T lymphocytes by adenosine-protein kinase A type I signaling. Cancer Res. (2007) 67:5949–56. 10.1158/0008-5472.CAN-06-4249 17575165

[42] Liu B Qu L Yan S. Cyclooxygenase-2 promotes tumor growth and suppresses tumor immunity. Cancer Cell Int. (2015) 15:106. 10.1186/s12935-015-0260-7 26549987 PMC4635545

[43] Majumder M Nandi P Omar A Ugwuagbo KC Lala PK. EP4 as a Therapeutic target for aggressive human breast cancer. Int J Mol Sci. (2018) 19:E1019. 10.3390/ijms19041019 29596308 PMC5979567

[44] Bozkulak EC Weinmaster G. Selective use of ADAM10 and ADAM17 in activation of Notch1 signaling. Mol Cell Biol. (2009) 29:5679–95. 10.1128/MCB.00406-09 19704010 PMC2772745

[45] Kveiborg M Instrell R Rowlands C Howell M Parker PJ. PKCα and PKCδ regulate ADAM17-mediated ectodomain shedding of heparin binding-EGF through separate pathways. PLoS ONE (2011) 6:e17168. 10.1371/journal.pone.0017168 21386996 PMC3046143

[46] Thorp E Vaisar T Subramanian M Mautner L Blobel C Tabas I. Shedding of the Mer tyrosine kinase receptor is mediated by ADAM17 protein through a pathway involving reactive oxygen species, protein kinase Cδ, and p38 mitogen-activated protein kinase (MAPK). J Biol Chem. (2011) 286:33335–44. 10.1074/jbc.M111.263020 21828049 PMC3190938

[47] Isakov N Altman A. Protein kinase C(theta) in T cell activation. Annu Rev Immunol. (2002) 20:761–94. 10.1146/annurev.immunol.20.100301.064807 11861617

[48] Nath PR Isakov N PKCθ-regulated signaling in health and disease. Biochem Soc Trans. (2014) 42:1484–9. 10.1042/BST20140180 25399558

[49] Steinbuck MP Winandy S. A review of notch processing with new insights into ligand-independent notch signaling in T-cells. Front Immunol. (2018) 9:1230. 10.3389/fimmu.2018.01230 29910816 PMC5992298

[50] Moretti J Brou C. Ubiquitinations in the notch signaling pathway. Int J Mol Sci. (2013) 14:6359–81. 10.3390/ijms14036359 23519106 PMC3634445

[51] Platonova N Manzo T Mirandola L Colombo M Calzavara E Vigolo E. PI3K/AKT signaling inhibits NOTCH1 lysosome-mediated degradation. Genes Chrom Cancer (2015) 54:516–26. 10.1002/gcc.22264 26052821

[52] Checquolo S Palermo R Cialfi S Ferrara G Oliviero C Talora C. Differential subcellular localization regulates c-Cbl E3 ligase activity upon Notch3 protein in T-cell leukemia. Oncogene (2010) 29:1463–74. 10.1038/onc.2009.446 19966856

[53] Sitkovsky MV Lukashev D Apasov S Kojima H Koshiba M Caldwell C. Physiological control of immune response and inflammatory tissue damage by hypoxia-inducible factors and adenosine A2A receptors. Annu Rev Immunol. (2004) 22:657–82. 10.1146/annurev.immunol.22.012703.104731 15032592

[54] Auderset F Schuster S Coutaz M Koch U Desgranges F Merck E. Redundant Notch1 and Notch2 signaling is necessary for IFNγ secretion by T helper 1 cells during infection with Leishmania major. PLoS Pathog. (2012) 8:e1002560. 10.1371/journal.ppat.1002560 22396647 PMC3291656

