# Corrigendum: Adenosine A2A Receptor Stimulation Inhibits TCR-Induced Notch1 Activation in CD8+T-Cells

**DOI:** 10.3389/fimmu.2019.00935

**Published:** 2019-05-03

**Authors:** Claudia Sorrentino, Fokhrul Hossain, Paulo C. Rodriguez, Rosa A. Sierra, Antonio Pannuti, Stephen Hatfield, Barbara A. Osborne, Lisa M. Minter, Lucio Miele, Silvana Morello

**Affiliations:** ^1^Department of Pharmacy, University of Salerno, Fisciano, Italy; ^2^Department of Genetics and Stanley S. Scott Cancer Center, Louisiana State University Health Sciences Center, New Orleans, LA, United States; ^3^H. L. Moffitt Comprehensive Cancer Center, Tampa, FL, United States; ^4^New England Inflammation and Tissue Protection Institute, Northeastern University, Boston, MA, United States; ^5^Department of Veterinary and Animal Sciences, University of Massachusetts, Amherst, MA, United States

**Keywords:** adenosine, A2A adenosine receptor (A2AR), Notch1, CD8+T-cells, immunosuppression

“Stephen Hatfield” was not included as an author in the published article. The corrected Author Contributions Statement appears below. The authors apologize for this error and state that this does not change the scientific conclusions of the article in any way. The original article has been updated.

## Author Contributions

SM and LM conceived the study, supervised experiments, and prepared the final version of the manuscript. CS, FH, AP, and RS performed experiments and interpreted their results. PR supervised experiments in Notch1IC-transgenic T-cells, which were performed by RS. SH provided *A2AR*−/− T cells and was responsible for maintaining adenosine receptor transgenic mouse lines. BO and LMM provided expertise on TCR signaling and PKC activity, contributed to result interpretation, reviewed and edited the final version of the manuscript.

