# Supplementary figures and images for: Adenosine A2A Receptor Stimulation Inhibits TCR-Induced Notch1 Activation in CD8+T-Cells
Source: Front Immunol. 2019 Feb 7;10:162. doi: 10.3389/fimmu.2019.00162 (PMC6374329; doi:10.3389/fimmu.2019.00162)

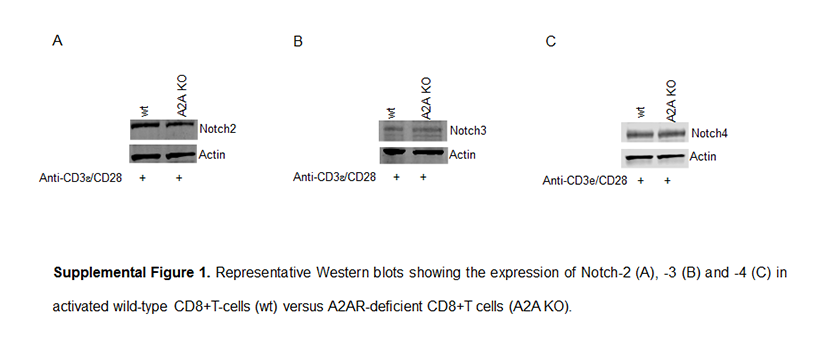

Supplement: Supplementary file 1 [file Image_1.TIF]
